# Supplementary material for: Investigating the potential of the secretome of mesenchymal stem cells derived from sickle cell disease patients
Source: PLoS One. 2019 Oct 30;14(10):e0222093. doi: 10.1371/journal.pone.0222093 (PMC6821040; doi:10.1371/journal.pone.0222093)
Supplement: S1 Table — (PDF) [file pone.0222093.s001.pdf]

S1_TABLE

Primer sequences used for quantitative reverse transcriptase–polymerase chain reaction gene expression analysis

Gene	Forward	Reverse
TGFBI	CATCAGCTACGAGTGCTGTC	GTGTACAGCTGAGTGGTGGT
COL4A4	AGATAAGGGTCCAACTGGTGT	ACCTTTAACGGCACCTAAAATGA
COL1A1	CACACGTCTCGGTCATGGTA	AAGAGGAAGGCCAAGTCGAG
FGF1	GATGGGACAAGGGACAGGAG	CGTATAAAAGCCCGTCGGTG
HGF	GCCTCTGGTTCCCCTTCAAT	GCTGCGTCCTTTACCAATGA
CXCL8	CAGGAAGAAACCACCGGAAG	TCTTTAGCACTCCTTGGCAAAA
SDF1	ATGAACGCCAAGGTCGTG	GGCTTTCGAAGAATCGGCAT
THBS1	TTGCCACAGCTCGTAGAACA	CAATGCCACAGTTCCTGATG
PCOLCE	GGAGTGCATCTGGACCATAAC	GAAGACTCGGAATGAGAGGGA
SPARC	GCTGGATGAGAACAACACCC	GCAGGAAGAGTCGAAGGTCT
MMP2	AGCTCCCGGAAAAGATTGATG	CAGGGTGCTGGCTGAGTAGAT
GAPDH	ACCCACTCCTCCACCTTTGA	CTGTTGCTGTAGCCAAATTCGT
HPRT1	GAAGTCTTGCTCGAGATGTGA	TCCAGCAGGTCAGCAAAGAAT
RN18S1	GACTTCACGCAAGCCTATGAC	CTGTGATGCCCTTAGATGTCTG

Abbreviations: TGFB1, transforming growth factor beta induced; COL4A4, collagen type IV alpha 4 chain; COL1A1, collagen type I alpha 1 chain; FGF1,fibroblast growth factor 1; HGF, hepatocyte growth factor; CXCL8, C-X-C motif chemokine ligand 8; SDF1a, stromal cell-derived factor-1, THBS1, thrombospondin 1 (THBS1); PCOLCE, procollagen C-endopeptidase enhancer; SPARC, secreted protein acidic and cysteine rich, MMP2, matrix metallopeptidase 2, GAPDH Glyceraldehyde 3-phosphate dehydrogenase; HPRT1: hypoxanthine phosphoribosyltransferase 1; RN18S1: RNA, 18S ribosomal 5 COL1A1 collagen, type I, alpha 1;

TABLE 2

	Normoxia	Hypoxia
CD14	6,9 ±1,5	0,4 ±0,5
CD31	0,6 ±1,0	1,8 ±1,2
CD34	4,0 ±0,6	0,7 ±0,8
CD29	93,5 ±4,5	87,2 ±7,1
CD73	60,0 ±4,0	58,6 ±6,4
CD90	96,9 ±3,5	90,6 ±4,2
CD105	96,2 ±8,6	81,4 ±6,9

Flow cytometry analysis of BMSC cultures isolated from SCD patients. BMSC were isolated, expanded and preconditioned in normoxic or hypoxic conditions. No significant differences in surface marker expression were found between normoxia or hypoxia groups. Data represent mean ±SD of three different experiments.
