# Supplementary material for: Investigating the potential of the secretome of mesenchymal stem cells derived from sickle cell disease patients
Source: PLoS One. 2019 Oct 30;14(10):e0222093. doi: 10.1371/journal.pone.0222093 (PMC6821040; doi:10.1371/journal.pone.0222093)
Supplement: S2 Table — BMSC were isolated, expanded and preconditioned in normoxic or hypoxic conditions for 48hs. No significant differences in surface marker expression were found between normoxia or hypoxia groups. Data represent mean ±SD of three independent experiments. (PDF) [file pone.0222093.s002.pdf]

S2_TABLE

	Normoxia	Hypoxia
CD14	6,9 ±1,5	0,4 ±0,5
CD31	0,6 ±1,0	1,8 ±1,2
CD34	4,0 ±0,6	0,7 ±0,8
CD29	93,5 ±4,5	87,2 ±7,1
CD73	60,0 ±4,0	58,6 ±6,4
CD90	96,9 ±3,5	90,6 ±4,2
CD105	96,2 ±8,6	81,4 ±6,9

Profiling of cell surface markers - Flow cytometry analysis of BMSC cultures isolated from SCD patients. BMSC were isolated, expanded and preconditioned in normoxic or hypoxic conditions. No significant differences in surface marker expression were found between normoxia or hypoxia groups. Data represent mean ±SD of three different experiments.
