# Supplementary material for: Investigating the potential of the secretome of mesenchymal stem cells derived from sickle cell disease patients
Source: PLoS One. 2019 Oct 30;14(10):e0222093. doi: 10.1371/journal.pone.0222093 (PMC6821040; doi:10.1371/journal.pone.0222093)
Supplement: S3 Table — (PDF) [file pone.0222093.s003.pdf]

S3 Table

Hematologic characteristics of SCD patients

Case n°	Gender	Age, years	Hemoglobin genotype	Hematocrit (%)	Hemoglobin (g/dL)	Chronic blood transfusions	Hydroxyurea treatment	Chronic pain	Taking oral opioid	Complication
1	F	18	HbSS	27.0	8.1	None	None	None	None	ONFH
2	M	26	HbSS	24.0	9.0	None	None	Yes	Yes	ONFH
3	M	42	HbSS	31.2	10.4	None	None	Yes	Yes	ONFH
4	F	50	HbSS	27.0	9.5	None	None	Yes	Yes	ONFH
5	F	18	HbSS	26.1	9.2	None	None	Yes	Yes	ONFH
6	M	26	HbSS	33.7	11.5	None	Yes	Yes	None	ONFH
7	F	31	HbSS	30.0	9.2	None	None	Yes	None	ONFH
8	M	33	HbSS	28.0	8.6	None	Yes	Yes	Yes	ONFH
9	M	25	HbSS	27.0	9.2	None	Yes	Yes	Yes	ONFH

Abbreviation: ONFH, osteonecrosis femoral head

HbSS: Individuals homozygous for the *HBB* glu6val mutation

Clinical and laboratory results obtained when patients were at steady state, without evidence of acute infection or pain crisis
