# Supplementary material for: Investigating the potential of the secretome of mesenchymal stem cells derived from sickle cell disease patients
Source: PLoS One. 2019 Oct 30;14(10):e0222093. doi: 10.1371/journal.pone.0222093 (PMC6821040; doi:10.1371/journal.pone.0222093)
Supplement: Attachment — Submitted filename: Minor revision requirements.pdf [file pone.0222093.s004.pdf]

In this study, Ribeiro et al. investigated the angiogenesis and tissue repair potential from the secretomes of mesenchymal stem cells collected from sickle cell disease patients, with some interesting results regarding the use of hypoxic preconditioning. Overall, this manuscript is straightforward, well-written, and the authors appear to utilize some widely-used techniques appropriately. The conclusions seem to be supported by the data shown and all ethics statements appear to be in order, making this a strong candidate for publication. But I do have some minor questions and considerations:

1) It's not stated from where the primers used in the Real Time qPCR analysis came from. Were they designed by the team or obtained from a source? If they were obtained please state the source and provide a link, if possible. Also, please include the gene symbol, gene name and accession number if possible in table 1.

2) Please standardize all the expressions in Latin (*in vitro*, *in vivo*, etc.), some are in italic but most aren't, making it a bit confusing sometimes (like line 377). Also gene names.

3) It is not clear why the stated passages were used in the experiments (5 passages (which ones?) for the HUVECs, passages 3-6 for all the BMSCs experiments, etc), please justify the election of the passages used. Did you perform growth curve and/or Doubling Time assays?

4) Only one assay is reported as being performed in triplicate. Please clarify if all of the experiments were done this way or not.
