# Supplementary material for: Investigating the potential of the secretome of mesenchymal stem cells derived from sickle cell disease patients
Source: PLoS One. 2019 Oct 30;14(10):e0222093. doi: 10.1371/journal.pone.0222093 (PMC6821040; doi:10.1371/journal.pone.0222093)
Supplement: Attachment — Submitted filename: Response to reviwers_PLos ONE_23July_Revision.pdf [file pone.0222093.s005.pdf]

Point by point response to Reviewers' comments (our responses are in italic and highlighted)

Comment. We thank the reviewers for the constructive criticism. We have addressed the major concerns of the reviewers, which have improved our manuscript. We also present the analyses and findings in a clearer manner according to the suggestions of the reviewers.

Reviewer #1 (R#1):

R#1: The Manuscript named "Investigating the potential of the secretome of mesenchymal stem cells derived from sickle cell disease patients" presented is well structured and designed. I believe that the manuscript has potential to be publish after some clarifications and changes

Answer *Answer: We thank the reviewer for the accurate summary of our results and the positive comments about our study.*

R#1: Why did the authors affirmed that they tested a hypothetically molecules? Why did the authors test unsure molecules?

Line 25: "in addition to the expression of paracrine molecules that hypothetically contribute to angiogenesis and skin regeneration"

Answer: We thank the reviewer for pointing this out.

We have reworded the abstract line 25 to more accurately reflect our data.

R#1: Line 35: Please review this statement: "In sum, culturing under hypoxic conditions produced profound effects on the BMSC secretome, as demonstrated by the expression of trophic paracrine factors involved in angiogenesis and skin regeneration".

Answer: We thank the reviewer for the comment.

We have rephrased the sentence to more accurately reflect our conclusion (line 35).

R#1: Line 59: "and promotes tissue healing and the formation of new blood vessels."

Change and promotes for ", promote". And the formation for "formation

Answer: Corrected. We thank the reviewer for pointing this out and apologies for the incorrect grammar.

R#1: Line 60: the main bioactive factors change for "important bioactive factor"

Av. Reitor Miguel Calmon, s/n – Vale do Canela –Salvador / Bahia CEP: 40110-902
Tel.: 55 - 71 - 3283-8918 - Fax.: 55 - 71 - 3283-8894

Answer: Corrected. We have changed the sentence (line 59) accordingly.

R#1: Line 63: Please review this statement: "design highly potent proangiogenic MSC-based cell therapies". The word design didn't fit well with the meaning.

Answer: We thank the reviewer for the comment.

We have rephrased the sentence (line 62) to more accurately reflect our conclusion.

R#1: Line 66 the gap: However, in SCD, 66 the key factors secreted by BMSCs that possess the potential to promote angiogenesis and tissue repair have not been identified to date.

Answer: Corrected. We thank the reviewer for pointing this out and apologies for the incorrect punctuation.

R#1: Line 78: Please add the reference for" The conditioned medium derived from the BMSC culture has been reported to serve multiple positive functions in tissue regeneration".

Answer: References added (line 78). We thank the reviewer for pointing this out.

R#1: Line 82: Please add the references for "Although numerous studies using BMSCs and their conditioned mediums as potential therapeutic agents have been published".

Answer: References added. We thank the reviewer for pointing this out.

R#1: Line 137: "The cells and the CM were then collected and processed at 3200 rpm for 20 min at 4° C, and kept at -70° C until use".

Was 0.5% oxygen for 48h enough to really see a hypoxic result?

Could -70o C affect the results?

When the authors collected CM, was it filtered?

Did the authors remove extracellular vesicles (which are bioactive vesicles) from CM?

Answer: We thank the reviewer for the questions.

*We have performed a hypoxic preconditioning protocol as previously described [XX]. According to the original article, a short-term physiological hypoxia increases the expression and secretion of angiogenic factors in mesenchymal stem cells (MSC) while potentiates its therapeutic functions (*Overath JM et al., Short-term preconditioning

enhances the therapeutic potential of adipose-derived stromal/stem cell-conditioned medium in cisplatin-induced acute kidney injury. Exp Cell Res. 2016 Mar 15;342(2):175-83). *Other studies also have shown that a single in vitro exposure of MSCs to hypoxia (0.5% O_2) significantly increased both the transcriptional and translational level of proangiogenic trophic factors* (Antebi B et al., Short-term physiological hypoxia potentiates the therapeutic function of mesenchymal stem cells. Stem Cell Res Ther. 2018 Oct 11;9(1):265).

To make it clear how we performed for conditioned medium collection and storage, we added a reference with the procedures previously described (Edwards SS et al., Functional analysis reveals angiogenic potential of human mesenchymal stem cells from Wharton's jelly in dermal regeneration. Angiogenesis. 2014;17(4):851-66.). *The* collected CM was not filtered *and the* extracellular vesicles were not removed. We also rephrased the sentence to make more accurate our data *(line 139, p.6).*

R#1: Line 150: “Relative mRNA expression of the target genes”

Was these mRNA isolated from CM or BMSC?

Answer: We thank the reviewer for pointing this out. mRNAs were isolated from BMSC. *We have added new sentences on* the manuscript M&M to clarify this point at p.7 (line 153).

R#1: Line 190: After a 1hour incubation period at 37 o C, the angioreactors were subcutaneously implanted into the dorsal flanks of 8 week-old female C57/BL6 mice.

How many animals were used?

Answer: We thank the reviewer for pointing this out. We have described the number of animals in these sets of experiments in the revised manuscript p. 9 (line 200).

R#1: Line 281: In “were highly positive for surface” Please add the percentage of each marker.

Answer: We thank the reviewer for pointing this out. We have included the percentage of each marker in the revised supporting Table 2, p.13.

R#1: How the authors did decide the secretome concentration/quantity for the in vivo study?

Answer: We thank the reviewer for this question. We have determined total protein in CM before injection. For in vivo studies we followed a *previously described protocol with the*

*measured amount of total protein (*Chen L et ., Conditioned Medium from Hypoxic Bone Marrow-Derived Mesenchymal Stem Cells Enhances Wound Healing in Mice. PLoS One. 2014 Apr 29;9(4):e96161). *We have added new sentences on line 140 and line 235 to make it clear.*

R#1: The results sections are lined with the M & M.

Answer: Corrected. *We thank the reviewer for the comment.*

R#1: Fig 3B add the STDEV or STD error on the graph.

Answer: Corrected. STDEV added to the graph on the Fig 3B. *We thank the reviewer for pointing this out.*

R#1: Line 460: I n "Recent studies ….", please add more references to this affirmative.

Answer: References added to this sentence in the revised discussion (Line 478). We thank the reviewer for pointing this out.

Other points:

R#1: In general, the authors performed a great study with very interesting results. But it is unclear if they considered in the M & M the proteins contained in the extracellular vesicles as part of the analyzed proteins or not. I suggest to the authors to let clear this point in the M & M.

Answer: We thank the reviewer for the positive comments and question. Secretome is a collective term and consists of extracellular vesicles (exosomes, microvesicles, membrane particles) and small soluble proteins (trophic factors). In this study we have investigated the total secretome rather than either soluble components or vesicular elements. We understand that both of these components may be capable of independently trigger regeneration and repair, but it was not the purpose of this manuscript. We have rephrased our M&M section (line 136-140) to make it clear and *to more accurately reflect our data*.

R#1: There are several studies indicating that extracellular vesicles have angiogenesis function. In the discussion the authors used the secretome as a general word to describe the compounds from CM, I believe that could be better to use the same concept in the material and methods. The authors could discuss better what is in secretome, and why to use a general cell secretion instead of only proteins and/or only extracellular vesicles?

Answer: We thank the reviewer for pointing this out and we discuss this in the revised manuscript p. 19-20. In this study, we were particularly interested in the effects of the total ”undivided” cell secretome, without separation or purification steps, mainly because it is more economical, more practical, and less time-consuming for clinical application. Also, use of the whole secretome avoids processing methods that can influence paracrine factor concentration and prevalence.

Reviewer #2 (R#2):

R#2: In this study everything seems to be according the questions above. The manuscript is presented in standard and correct English, all the experiments appear to have been done appropriately and the conclusion is supported by the data presented.

Answer: We thank the reviewer for the positive comments about our study.

Minor revision requirements

In this study, Ribeiro et al. investigated the angiogenesis and tissue repair potential from the secretomes of mesenchymal stem cells collected from sickle cell disease patients, with some interesting results regarding the use of hypoxic preconditioning. Overall, this manuscript is straightforward, well-written, and the authors appear to utilize some widely-used techniques appropriately. The conclusions seem to be supported by the data shown and all ethics statements appear to be in order, making this a strong candidate for publication.

Answer: We thank the reviewer for the accurate summary of our results and the positive comments about our study.

R#2: But I do have some minor questions and considerations:

1) It’s not stated from where the primers used in the Real Time qPCR analysis came from. Were they designed by the team or obtained from a source? If they were obtained please state the source and provide a link, if possible. Also, please include the gene symbol, gene name and accession number if possible in table 1.

Answer: We thank the reviewer for pointing this out. The team designed primer sequences. Primer sequence, gene symbol and gene name were included on Supporting Table 1.

2) Please standardize all the expressions in Latin (in vitro , in vivo , etc.), some are in italic but most aren't, making it a bit confusing sometimes (like line 377). Also gene names.

Answer: Corrected. We thank the reviewer for pointing this out.

3) It is not clear why the stated passages were used in the experiments at #5 passages (which ones?) for the HUVECs, passages 3-6 for all the BMSCs experiments, etc), please justify the election of the passages used. Did you perform growth curve and/or Doubling Time assays?

Answer: We thank the reviewer for the questions. The effects of serial cell passaging on cultured HUVEC have been previously investigated. As many primary cells, HUVEC are very sensitive to long-term culture and after 5 passages they lose their typical "cobblestone" morphology and angiogenic sprouting (tubule forming) efficiency (Nakatsu MN, Hughes CC. An optimized three-dimensional in vitro model for the analysis of angiogenesis. Methods Enzymol. 2008;443:65-82).

Similar situation occurs with BMSC. The International Society for Cellular Therapy (ISCT) has established the minimal criteria for defining multipotent bone marrow mesenchymal stromal cells (BMSC) (see Dominici M et al., Minimal criteria for defining multipotent mesenchymal stromal cells. The International Society for Cellular Therapy position statement. Cytotherapy. 2006. 8(4):315-7). To avoid substantial batch-to-batch variation in phenotype and function, the ISCT has recommended working with low passage BMSCs (Third to six passages). In our experience and the others, BMSCs expanded in FBS-supplemented media undergo a transition from predominately spindle-shaped at early passages to larger fried-egg-shaped the longer they are maintained in culture (Agata, H. et al. Feasibility and efficacy of bone tissue engineering using human bone marrow stromal cells cultivated in serum-free conditions. Biochem. Biophys. Res. Commun. 2009. 382, 353–358; Daltro et al., Efficacy of autologous stem cell-based therapy for osteonecrosis of the femoral head in sickle cell disease: a five-year follow-up study. Stem Cell Res Ther. 2015. 29;6:110).

4) Only one assay is reported as being performed in triplicate. Please clarify if all of the experiments were done this way or not.

Answer: Corrected. We thank the reviewer for pointing this out. All assays were performed at least in triplicate and independent experiments were repeated three times. We have rewritten our M&M section on p.11.

Av. Reitor Miguel Calmon, s/n – Vale do Canela –Salvador / Bahia CEP: 40110-902
Tel.: 55 - 71 - 3283-8918 - Fax.: 55 - 71 - 3283-8894
